# Smart Rings in Remote Monitoring at Home: Older Patients' Perceptions and Expectations

**DOI:** 10.1111/hex.70407

**Published:** 2025-09-01

**Authors:** Melika Azim Zadegan, Eeva Aromaa, Tero Montonen, Rosa Sahlström, Päivi Eriksson, Ville Leinonen

**Affiliations:** ^1^ Business School University of Eastern Finland Kuopio Finland; ^2^ Neurosurgery, Institute of Clinical Medicine University of Eastern Finland Kuopio Finland; ^3^ Department of Neurosurgery NeuroCenter, Kuopio University Hospital Kuopio Finland

**Keywords:** aged, digital health, interview, patients, qualitative research, remote patient monitoring, wearable electronic devices

## Abstract

**Background:**

There is a limited understanding of how older patients perceive the potential of using wearables for remote monitoring (RM) at home. Exploring patients' perspectives prior to the implementation of wearables in healthcare can offer valuable insights into what may enhance the effectiveness and outcomes of RM.

**Objectives:**

The objective of this study was to explore the expectations of older patients with a susceptible chronic illness towards prospective use of the Oura Ring for health monitoring at home. Unlike previous research, which primarily addresses patients' experiences during or after RM has taken place, we explored their perspective before the use.

**Methods:**

Healthcare professionals identified the 10 study participants among those who were undergoing a diagnostic procedure for the chronic neurological disease iNPH at the Kuopio University Hospital in Finland. All invited patients consented to participate. Qualitative interview data were analysed using reflexive thematic analysis by Braun and Clarke.

**Findings:**

Patients expected Oura to support both health and broader well‐being through self‐tracking and proactive health management, presenting themselves as active contributors to their care. Being monitored by healthcare professionals or caregivers was viewed as reassuring, mirroring the increasing digitalisation of healthcare and a reduction in in‐person contact.

**Conclusion:**

The findings highlight the relevance of approaching older patients with chronic conditions to gauge interest in home‐based RM. This emphasises involving the patient perspective early on in RM processes, including decisions about the specific solutions to be used.

**Patient or Public Contribution:**

The second author is a trained patient expert by experience who ensured that the analysis and the findings were grounded in authentic patient perspectives. The findings were shared with the study participants in the form of an information leaflet, the quality of which was ensured by the expert by experience.

## 1 Introduction

Remote monitoring (RM) solutions are being adopted as part of a broader shift towards digital health, with increasing emphasis on home‐based care, ranging from cancer [1] to orthopaedic surgery [2]. RM enables healthcare professionals (HCPs) to monitor patients remotely in their homes, transmitting their digital health data to clinical sites [3, 4]. At the same time, consumer wearables, such as wristbands and smart rings, are becoming more familiar among increasingly diverse user groups, including older adults [5].

Interest in wearables grew among researchers and HCPs during the Covid‐19 pandemic due to their potential as a user‐friendly solution for RM in healthcare settings [6]. Wearables continuously monitor several physiological metrics, such as heart rate, body temperature and sleep metrics, in real time [7]. Thus, wearables could constitute user‐friendly technology for RM at home [8, 9], particularly for older adults with chronic illnesses who require continuous care [10, 11].

Research has underscored the general importance of exploring the patient's perspective on digital health solutions in rapidly ageing societies [12], highlighting the need to involve patients in the early phases of digital health implementation [13], including the use of wearables [14, 15]. Prior studies on wearables have mostly focused on younger adults, leaving a gap in our understanding of older adults' and older patients' perspectives on their use [5, 9, 16, 17]. Relatively little is known about older adults' interest in wearables or to what extent older patients with chronic conditions are willing to use them [18, 19].

While some studies have shown low rates of wearable use among older adults [20, 21, 22], others have identified an interest in this group in using simple, functional and affordable wearables [11, 17]. Research has also found that older adults are more likely to reject wearables or stop using them if they do not perceive immediate value in them or if the wearables are not integrated into their daily routines [17, 23]. However, self‐tracking one's well‐being with wearables has been reported as the most expected use of technology among older adults [11, 21].

While older adults' interest in wearables seems to be changing, it is fruitful to study older patients' expectations regarding the prospective use of a specific wearable in home‐based RM. Older patients with chronic illnesses living at home are a rapidly growing group [12, 24, 25], which is why their expectations should be studied. Involving older patients and incorporating their views early in the RM process would strengthen their voice in this matter [4]. Furthermore, involving older patients in digital health planning and decision‐making processes is important because it may enhance acceptance, long‐term engagement and satisfaction with home care [13].

In this study, we examined older patients' perceptions and expectations of a specific wearable, a Finnish‐designed smart ring called the Oura Ring (hereafter Oura). The study participants were undergoing the diagnostic procedure of a chronic neurological illness, idiopathic normal pressure hydrocephalus (iNPH) disease, at the Kuopio University Hospital (KUH), located in a city within the sparsely populated North Savo region in Finland. This disease causes changes in patients' motor symptoms, which can be easily measured remotely, which is why this patient group would benefit from home‐based RM [26, 27]. The Finnish population is rapidly ageing, but mostly lives at home and is increasingly offered digital health services [12]. This makes Finland an interesting context for this study.

Our research question was as follows: *How do older patients perceive the potential use of Oura for RM at home, and what do they expect from it?* By addressing this question, we provide new knowledge of the benefits and challenges associated with a specific wearable as a potential solution for home‐based RM.

## 2 Methods

### 2.1 Study Design

The Research Ethics Board of the North Savo Hospital District was responsible for the pre‐evaluation of research conducted at KUH and approved the study (Approval number: 276/2016 (5/2008), with approval updated on 22 June 2021). Authors R.S and V.L., affiliated with KUH at the time of data collection, identified the study participants from referrals to the neurosurgery outpatient clinic for a diagnostic procedure. The key selection criteria were that the participants would likely benefit from home‐based RM, that they represented a diverse group in terms of age, gender and place of residence, and that they had no prior experience with Oura. No other criterion related to technology use was included. A neurosurgery professional evaluated each participant's cognitive orientation to ensure they understood the study. No patients were excluded based on this assessment.

All invited patients gave their consent to participate. The sample included roughly equal numbers of participants in terms of gender, age categories and place of residence. A pilot interview was conducted to check the accessibility and clarity of the short interview questions, which were designed to avoid excessively disrupting the participants' daily routines. No major modifications were needed, and the pilot interview was included in the study sample of 10 patients.

Between December 2022 and October 2023, 10 patients aged 64–80 were interviewed in Finnish, either face‐to‐face at an outpatient clinic of KUH or by phone by a HCP they knew (the author R.S.), which contributed to an atmosphere of trust. The interview started with a short section on prior technology use. The main part of the interview, including open‐ended thematic questions, focused on home‐based RM, the introduction of Oura and expectations toward its potential use. The interviews were transcribed verbatim and translated into English by a professional.

The participants spent between 10 and 20 min providing their insights. The two shortest interviews, lasting 10 and 12 min, were with those most hesitant towards RM. The interviewer encouraged the participants to openly share their insights on RM, but the brevity of the interaction may have limited the richness and nuance of the data. On the other hand, the last interviews provided little new insight, which can be a sign of approaching data saturation.

### 2.2 Analysis

The analysis followed the principles of reflexive thematic analysis outlined by Braun and Clarke [28] and Byrne [29], in which semantic units, broader categories and main themes are identified from data. The trained patient expert by experience in the author team (E.A.) was involved in all phases of data analysis and manuscript writing.

In the first phase of the analysis, we familiarised ourselves with the data by reading all interviews several times. In the second phase, the initial coding generated semantic units that captured detailed meanings across the dataset. In the third phase, these semantic units were grouped into broader categories, each encompassing more general patterns. In the fourth phase, the broader categories were combined into four main themes, providing an overarching structure to frame and interpret the findings. Table 1 illustrates how descriptions of several semantic units were grouped into broader categories, and how broader categories were grouped into main themes. The iterative analysis drew on researcher triangulation, involving continuous discussion among the authors to crystallise the findings [30].

**Table 1:** From descriptions of semantic units to broader categories and main themes.

Broader category based on several semantic units	Main themes
Everyday fit and familiarity Descriptions of comfort, practicality and continuous wearability Descriptions of habituation, familiarity and positive adaptation	Oura's features as a ring
Everyday wearability concerns Descriptions of concerns regarding daily activities Descriptions of concerns regarding the ring's durability	
User‐friendly design Descriptions of ease and convenience of use	Oura functions
Reliable and secure monitoring Descriptions of data accuracy and measurement capabilities Descriptions of data security and Health emergency features	
Personal health awareness General interest in self‐tracking Sleep and recovery monitoring	Self‐tracking
Self‐tracking as a tool and source of motivation Activity and vital sign tracking Motivation and preventive health awareness	
Trust and data boundaries Willingness to share data with healthcare professionals	Monitoring by healthcare professionals
Professional monitoring Health metrics for healthcare professionals to monitor Monitoring practice and data privacy	

The study findings were shared with participants in collaboration with the KUH neurosurgery outpatient clinic, in the form of an information leaflet, the quality of which was ensured by our trained patient expert with experience (author E.A.).

## 3 Findings

The participants' socio‐demographic characteristics are provided in Table 2. Of the ten participants, nine used a smartphone, eight reported previous use of digital health devices (e.g., blood pressure monitor), and three had used a wearable before (e.g., fitness tracker). Eight out of ten participants expressed openness towards home‐based RM. Two were hesitant towards all digital devices and had less interest in RM and using Oura as part of it.

**Table 2:** Socio‐demographic characteristics of participants.

Participants' characteristics	n
Age range	
64–69 years	2
70–75 years	4
76–80 years	4
Gender	
Female	5
Male	5
Place of residence	
Urban area (city, municipal centre and suburbs)	6
Non‐urban area (rural and sparsely populated areas)	4
Living arrangement	
Living with a spouse	9
Living alone	1
Smartphone	
Using	9
Not using	1
Digital health device (e.g., blood pressure monitor)	
Using	8
Not using	2
Consumer wearable (e.g., fitness tracker)	
Using	3
Not using	7

**Figure 1:**
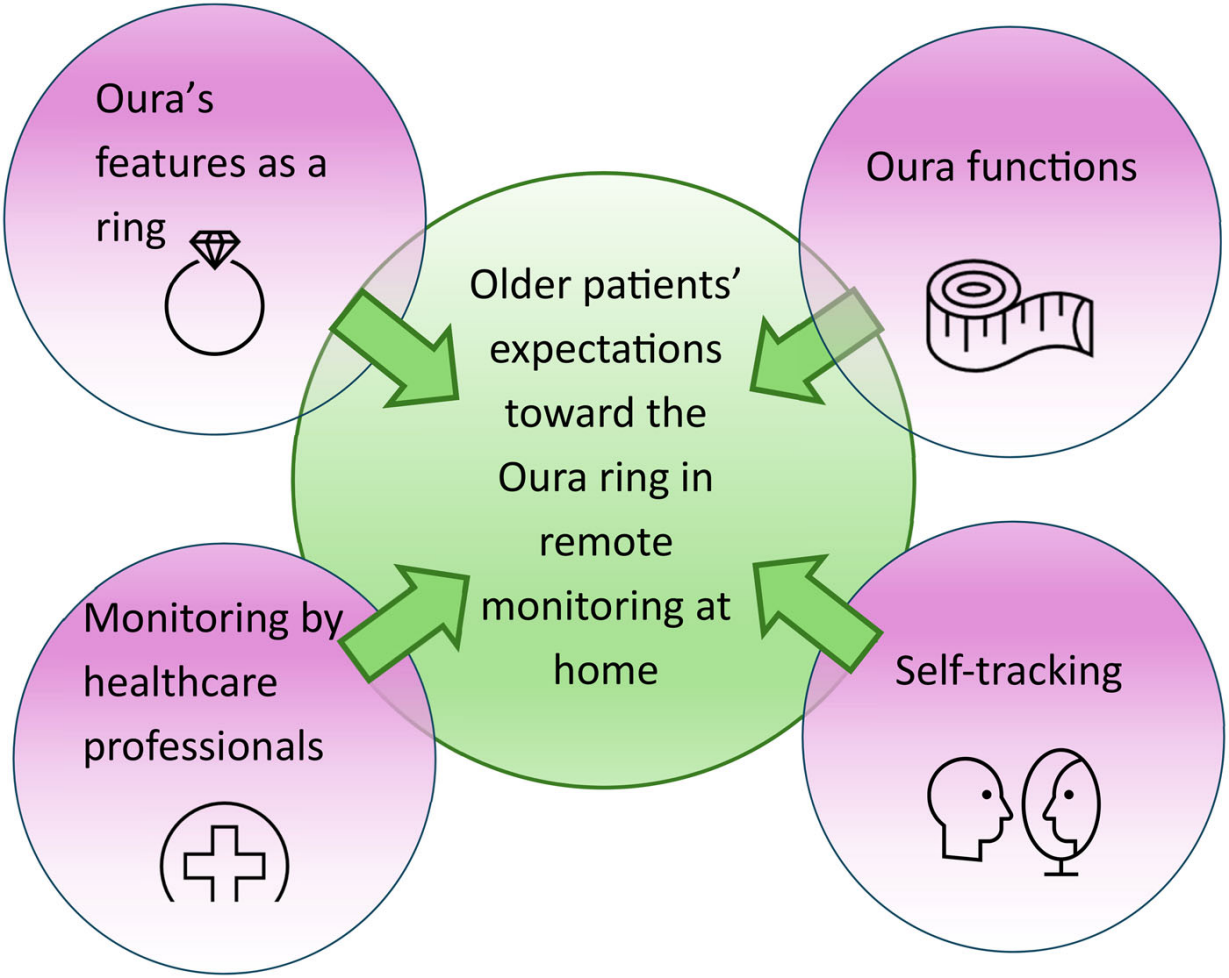
Thematic framework of the study. Arrows indicate how the main themes contribute to the research question.

The patient perspective and their more specific expectations centred on four main themes: (1) Oura's features as a ring, (2) Oura functions, (3) self‐tracking and (4) monitoring by HCPs. Together, these themes contribute to the proactive management of not only health but also broader well‐being. Figure 1 presents a thematic framework to help interpret these connections.

### 3.1 Oura's Features as a Ring

#### 3.1.1 Everyday Fit and Familiarity

The patients noted the practical benefits and comfort associated with wearing a ring continuously. Due to this, Oura was considered practical in everyday use, and the ease of its integration into daily life was seen as a major benefit. When comparing Oura with a wrist‐worn bracelet, the physical comfort of a ring was seen as a major difference, highlighting that Oura's smaller size and lighter design would make it more comfortable for daily use and less likely to irritate during sleep or exercise:

Well, in a way, it's more practical, as you have it there, on your finger. You won't … it's not there, like, in a way…. I'd wear one on my wrist, too, but it is a lot more unnoticeable, that ring; or in a way, as you have other rings, too, you won't be thinking about it like that … that it's there, measuring things or … if you can think that way.

Participant 6

*I guess the ring feels better to me because the bracelet made me sweaty sometimes, and it's quite big. So, I guess the ring would feel a bit [lighter]*.

Participant 7

While patients expressed confidence in their ability to use Oura, some noted that it might take some time to get used to one more ring on their finger. However, patients had an overall positive view of adaptation, suggesting that any initial discomfort caused by one more ring on their finger would be temporary and likely to be quickly resolved, and familiarity with wearing other rings eliminated the sense of inconvenience:

Well, of course it would be an extra ring there, on my finger so that, I've only had my wedding ring and well, now if there'd be another one, then I guess it would take some time to get used to it.

Participant 4

The discomfort that it causes on your finger, that doesn't matter at all as you get used to it in twenty‐four hours.

Participant 5

I'm used to wearing rings as it is, so I could wear another one.

Participant 7

#### 3.1.2 Everyday Wearability Concerns

Although patients' perceptions of Oura were generally positive, they raised some practical concerns about using it in everyday activities during which it might need to be removed, and they might forget to put it on their finger again:

Well, of course, this, whether you can go to the sauna with it or have a wash when wearing it and other things like that. So, if you have to take it off at times for some reason, will you remember to put it back on and…?

Participant 5

Patients also raised the concern that their existing habits might conflict with consistent Oura use, which could negatively impact the RM process:

Back when I used to wear actual rings, it was my habit that whenever I washed the dishes or worked or something, I removed them. I guess I would continue doing the same thing with that, so I guess that would be bad.

Participant 9

Patients raised concerns about Oura's durability and its resistance to environmental conditions during leisure activities. They expressed uncertainty about how it would perform during physical activities such as sports or when exposed to moisture and extreme temperatures, particularly during sauna use or in Finland's cold climate:

How about, well, is it resistant to bumps or something like that? If you think that you're engaging in sports, such as volleyball or something like that, do you have to take it off?

Participant 5

I don't know. What about if it gets wet? Is it protected in some way?

Participant 1

How about sauna and freezing temperatures?

Participant 1

### 3.2 Oura Functions

#### 3.2.1 User‐Friendly Design

Patients perceived Oura to be simple enough and effortless to use. Simplicity in basic functions and integration into daily life were seen as key conditions for its potential use in home‐based RM. The idea of having a single device for health monitoring was appreciated, and the value of not needing to interact with the device regularly for entering one's health data was noted as a key benefit:

Well, one of them is this: having just one device to do the measurements. So, with this one, you had this gadget on your belt at times, on your wrist at others. And with that one, it seems like you won't have to think about that…. It's easier.

Participant 1

Well, not having to wind it, like an old wristwatch or something. Or … press it fifteen past nine every morning and evening or … I mean, this easiness is the keyword here.

Participant 5

#### 3.2.2 Reliable and Secure Monitoring

Patients were curious about the kind of data Oura could collect, how accurate it would be and how the data would be accessed and interpreted. They questioned how a small, familiar object like a ring could reliably gather health data and produce accurate information for HCPs:

So, if it is just a normal ring, where do you get all that data from?

Participant 2

Some expressed uncertainty about what Oura actually tracks and how the results would be accessed or understood, reflecting a desire for clarity about the ring's measurement capabilities, the type of data it provides and whether patients can access it and if they can interpret the results themselves:

But … and then there is the thing with interpreting it, too, of course. So, I don't know what this Oura … what are all the things you can track with it?

Participant 4

Well, I don't know if I need to know that much about it. What … it's more like I either read about it myself or someone tells me what it's producing, which data.

Participant 5

How do you get the data out of it? Do you enter it via Bluetooth to your computer or something?

Participant 9

Patients noted the potential for Oura to support safety and well‐being through its alert functions. They imagined it playing an active role in detecting and responding to critical health situations, such as a patient's decreased activity, reflecting a hopeful view of Oura as not only a passive monitoring tool but also a device capable of providing real‐time alerts:

It could even sound an alarm if there are any critical changes in your activity level, or mostly that.

Participant 1

Patients also expressed concerns about data privacy, highlighting the importance of knowing who can access their personal health information and of maintaining control over who can access their personal health information:

Mostly security matters, as in how secured the data is, so it won't be given to parties to whom it shouldn't be given. So, information security is the thing. But I cannot think of anything else. I have no particular prejudices about it.

Participant 7

### 3.3 Self‐Tracking

#### 3.3.1 Personal Health Awareness

Patients were interested in tracking their own health metrics and viewed self‐tracking as a helpful way to stay informed about their health. They expressed broad interest in tracking various health indicators, including those beyond Oura's current capabilities, such as blood pressure, and considered self‐tracking to be both familiar and beneficial, particularly in relation to physical activity:

And all the things you can track with it, I'm interested in it all, so whether my heart or all that blood pressure.

Participant 6

Those who were familiar with wearables perceived evaluating the effects of physical activity on daily life using Oura as easy:

Since that's familiar stuff in that I think it's kind of the basic stuff that's always measured, steps and activity…. So, it was quite interesting to keep track of them. How your activity affects you daily. So, I only have positive thoughts about it.

Participant 7

Patients also inquired about the possibility of sharing health data with family members, such as children and spouses, suggesting that tracking could ease family members' concerns about their health conditions:

And I believe that our children may also be interested in seeing how I'm doing.

Participant 1

Patients were extremely interested in sleep tracking and recovery monitoring and were curious to know whether their experience of sleep aligned with reality. This reflected a desire to gain objective sleep data rather than relying on subjective feelings. Some patients even emphasised sleep monitoring as their most central issue:

Well, it's the activity, of course, and sleep is one of those things. I think that when I close my eyes in the evenings, I fall asleep after it and wake up in the morning. Is that really the case? I don't know.

Participant 1

Sleep. Sleep is maybe the main thing, and I feel like, of course, these interruptions of breathing as well. I have them every night, and like I said, after I retired, I've had this machine [a device that supports respiration] ever since then.

Participant 4

#### 3.3.2 Self‐Tracking as a Tool and Source of Motivation

Patients perceived the value of using Oura to track daily activity and vital signs. For many, health and activity tracking were associated with a sense of control and the potential for early detection of health concerns. For many, activity tracking was meaningful due to challenges in everyday physical activities, and some showed a broad interest in tracking vital signs and general health markers:

I have balance, problems with my balance and such, so … I could, of course, track my number of steps and activity.

Participant 4

Well, of course, my heart rate, consciousness and all these kinds of things are, like, and then the amount of physical activity and well…. In general, things related to health.

Participant 5

Some patients also noted that, as they have an intuitive sense of their daily activities, they have no use for Oura:

Well, I pretty much know the steps that I take every day.

Participant 3

Most patients viewed self‐tracking as a tool for RM and a source of motivation to increase physical activity and early detection of potential health issues, supporting preventive health behaviour. They described how health and activity data could act as motivators, helping them to recognise areas for improvement in their daily habits regarding their health. Some further highlighted the importance of identifying subtle warning signs before more serious symptoms arise:

So, it can really be … like inspire you more when you get to see, at times, what … what your results are or where there's room for development.

Participant 6

The interest is one thing, of course, and also, if there were any alarming signs, that would also be good to know. So, if there's something that's a bit alarming.

Participant 7

### 3.4 Monitoring by HCPs

#### 3.4.1 Trust and Data Boundaries

Patients were open to sharing their monitoring data with HCPs, seeing it as a natural part of the care received at the hospital outpatient clinic. They perceived the potential sharing of Oura data with HCPs as beneficial as long as the data remained accessible to only the patient's healthcare team:

I feel like it's part of this. I mean, you track these things anyway so … used to having these be, maybe not public information as such, but necessary for the people involved in [my] treatment.

Participant 1

The question is what it can actually do, for there are things that are interesting to a physician as well…. The physician wants to know about that stuff just as well.

Participant 7

#### 3.4.2 Professional Monitoring and Privacy

Patients had varying expectations regarding what data should be monitored by HCPs. While most of them had positive attitudes towards health metric monitoring, some expressed discomfort with continuous RM enabled by Oura. Sleep and vital sign tracking by HCPs were considered the most important, and patients noted that any changes in the monitoring data, whether the numbers were too high or too low, would be difficult to interpret on their own:

And of course, that sleep quality is an important one, too, so that…. So, these sorts of professionals could keep checking if my sleep quality is the way it should be.

Participant 2

Of course, well … I mean, for example … pulse is one of them. It tells you something when it gets higher, when it's gotten too high or when it's too low, or…. Sleep is also a thing that's difficult to track on your own.

Participant 5

On the other hand, not all patients were comfortable with the idea of constant RM throughout the day and night, with some describing it as potentially burdensome or emotionally uncomfortable:

Well, I don't know, as they're monitoring me anyway! I don't feel like that's my thing…. The monitoring [feels difficult], yes.… I don't think I would find it enjoyable at least.

Participant 10

Patients raised practical and ethical questions about how the monitoring data would be managed within the healthcare system. Concerns focused on data access, privacy boundaries and how monitoring would be handled in clinical settings:

Yeah, and the healthcare staff … who does that include? So, is it, like, shared nationally? Or is there some limited number of people monitoring the data?

Participant 4

Some wanted to know who could access their data, and some expressed curiosity about whether RM could reduce the need for in‐person clinical visits, indicating openness to RM with Oura if it leads to more efficient care but expressing uncertainty about how such processes would work in practice:

It goes there … [unclear] You probably won't have to visit a doctor there so much, so that if it, like, notifies whether something is wrong, or…

Participant 6

Questions were also raised about how actively and how often the data would be reviewed and whether HCPs would monitor the data in real time or at defined intervals:

How actively would the physician or nurse monitor it? Would they do it at certain intervals?

Participant 9

## 4 Discussion

### 4.1 Key Findings

This study explored the perceptions and expectations of older patients with a chronic condition towards the potential use of a specific wearable, Oura, in home‐based RM. Involving patients early on in digital health planning and decision‐making processes is important because it may enhance acceptance, long‐term engagement and satisfaction with care in the context of RM [13].

The first key finding is that, in addition to most patients being interested in RM, their expectations extended beyond Oura's features and functionalities, as they hoped it could support not only their health but also their overall well‐being. Patients expected Oura to enable proactive health and well‐being management, particularly by helping to detect rapid changes. Interested patients presented themselves as agentic and motivated individuals, both when it came to being monitored by HCPs and in self‐tracking [25, 31], provided that simple, user‐friendly solutions were available in healthcare [17, 19]. This key finding challenges earlier reports of limited interest in wearables among older adults [20, 22] and instead supports more recent evidence of older patients' engagement in health monitoring [11] and self‐tracking [21].

The second key finding highlights an underexplored motivational factor for RM engagement among older patients with chronic conditions. The possibility that HCPs and caregivers, such as children and spouses, could access patients' health and well‐being data was perceived as reassuring and motivating rather than problematic. This kind of oversight was expected to encourage healthier everyday decisions, such as increasing physical activity. The sense of ‘being watched’ through Oura was, by some, interpreted as a form of ‘being cared for’ and ‘being guided’ by HCPs, family members and caregivers. These findings resonate with the contextual features of Finnish society, where digital health solutions are widely implemented across many areas of life, including healthcare and well‐being [12]. The findings may also reflect the overall decline in face‐to‐face care within Finnish healthcare, a trend that disproportionately affects older adults [32].

An additional finding shows that while interested patients were generally open to sharing their RM data, the prospect of continuous monitoring raised concerns about privacy, personal boundaries and emotional discomfort. Patients posed questions about how RM would function in practice, expecting to be provided detailed information about data access, monitoring frequency and the potential impact on the need for in‐person clinical visits. As found by Baines et al. [13] and Villani et al. [33], technical features, uncertainties and privacy concerns were noted. In our study, however, patients' questions in the pre‐implementation phase indicated not only uncertainties but also a proactive orientation towards RM.

### 4.2 Strengths, Limitations and Future Research

A key strength of this study is its focus on the pre‐implementation phase of RM in the North Savo region of Finland, where older adults with chronic conditions commonly live at home, including in rural areas far from hospital clinics. Such a context, where the need for home‐based RM is potentially high, emphasises the importance of considering patients' expectations prior to implementation, ensuring they have a voice in the process. To our best knowledge, this study is the first in Finland to focus on older patients' perspectives on RM before implementation.

However, the study also has limitations. Most of the participants in this study lived with someone else (spouse, caregiver, etc.), which encourages other studies to include more patients living alone. Also, due to the small sample size and the brevity of the interviews, including more participants and conducting longer interviews could have enhanced the richness and depth of the findings, allowing for a broader and more nuanced understanding of the patient perspective.

Future research should focus on fostering broader patient involvement during the pre‐implementation phase of RM, when patient benefits and concerns can be considered in the planning process. Our findings encourage engaging not only patients alone but also caregivers and relevant HCPs to better understand how Oura could be meaningfully integrated into home care. Particular attention should be paid to the needs, capabilities and daily routines of patients to ensure solutions are accessible, acceptable and supportive of long‐term use.

### 4.3 Practical Implications

It is perhaps unsurprising that patients expressed a desire for Oura to support their overall well‐being, not just health or illness‐related concerns. Many of Oura's core features, such as sleep tracking, activity monitoring and stress indicators, are designed with everyday well‐being in mind. While healthcare systems often prioritise clinical indicators of disease, patients may value tools that reflect and respond to their lived experience of feeling well.

Nevertheless, our findings should encourage HCPs to explore the use of Oura as part of home care that supports not only health but also broader well‐being. Where appropriate, Oura could even serve as a practical alternative to medical devices, provided it produces the information needed and is accurate, secure and aligned with healthcare realities. On the other hand, whatever the solution in use, HCPs are regarded by patients as trusted sources of information and guidance related to RM and are frequently approached for support in these areas [13]. To fulfil this role, HCPs should be trained to address patient concerns, clearly describe RM processes and communicate openly about the benefits and challenges involved. HCPs should also communicate how patient data is used, including how and when patients receive information derived from their data, who communicates it, and what kind of follow‐up is expected. Feedback loops that allow patients to receive timely and relevant responses to their data can enhance motivation, especially when combined with educational support, coaching or shared decision‐making [34].

Finally, developers and decision‐makers should co‐design RM solutions with end users to ensure that they meet expectations for usability, emotional comfort and integration into everyday life [35]. As older patients express interest in sharing their health status also with caregivers, it should be considered whether this expectation can be acknowledged and supported through security features and data‐sharing settings. HCPs should also be attentive to the motivational potential of being ‘seen’ or ‘cared for’ through RM. Designing care pathways that incorporate both professional oversight and caregiver involvement, while safeguarding patient autonomy, may improve the acceptability and impact of RM.

## 5 Conclusion

This study demonstrated that older patients with chronic conditions are open to using Oura for home‐based RM. Patients valued simplicity, comfort and caregiver access to data, viewing Oura as a source of motivation rather than an intrusion. Although Oura was introduced as a tool for RM, patients easily envisioned its potential to go beyond the measurement of disease‐related symptoms. They saw Oura as providing relevant insights into their health and well‐being status, not only to themselves, but also to caregivers and HCPs. Hence, Oura was not perceived as a standalone technological or clinical add‐on to patients' everyday lives. Rather, it was seen as a component of home care, supporting both illness management and overall wellness. Overall, the findings underscore the importance of involving patients early in the design and implementation of RM as part of home care, something that does not always happen [13, 36].

## Author Contributions

**Melika Azim Zadegan:** conceptualization, formal analysis, investigation, methodology, project administration, validation, visualization, writing – original draft preparation, writing – review and editing. **Eeva Aromaa:** conceptualization, formal analysis, investigation, methodology, supervision, validation, visualization, writing – original draft preparation, writing – review and editing. **Tero Montonen:** conceptualization, formal analysis, investigation, methodology, validation, visualization, writing – original draft preparation, writing – review and editing. **Rosa Sahlström:** data curation, resources, writing – review and editing. **Päivi Eriksson:** conceptualization, funding acquisition, methodology, resources, supervision, writing – original draft preparation, writing – review and editing. **Ville Leinonen:** resources, supervision, writing – review and editing.

## Ethics Statement

This study was approved by the Research Ethics Board of the North Savo Hospital District, Finland (Approval number: 276/2016 (5/2008)), with approval updated on June 22, 2021. The study adhered to the ethical principles outlined in the Declaration of Helsinki and complied with the EU General Data Protection Regulation (GDPR).

## Consent

Informed consent was obtained from all patients participating in the study before their participation, including consent to participate and consent to publication.

## Conflicts of Interest

The authors declare no conflicts of interest.

## Data Availability

The data supporting the findings of this study are not publicly available due to privacy restrictions that protect the confidentiality of the participants.

## References

[1] V. LeBaron , “Challenges and Opportunities in Designing and Deploying Remote Health Monitoring Technology for Older Adults With Cancer,” Innovation in Aging 6, no. 6 (2022): 1–7. 10.1093/geroni/igac057 PMC9701055 36452048

[2] F. Moldovan and L. Moldovan , “An Innovative Assessment Framework for Remote Care in Orthopedics,” Healthcare 13, no. 7 (2025): 736. 40218034 10.3390/healthcare13070736 PMC11988397

[3] S. Y. Tan , J. Sumner , Y. Wang , and A. Wenjun Yip , “A Systematic Review of the Impacts of Remote Patient Monitoring (RPM) Interventions on Safety, Adherence, Quality‐of‐Life and Cost‐Related Outcomes,” NPJ Digital Medicine 7, no. 192 (2024): 192. 39025937 10.1038/s41746-024-01182-w PMC11258279

[4] R. C. Walker , A. Tong , K. Howard , and S. C. Palmer , “Patient Expectations and Experiences of Remote Monitoring for Chronic Diseases: Systematic Review and Thematic Synthesis of Qualitative Studies,” International Journal of Medical Informatics 124 (2019): 78–85. 30784430 10.1016/j.ijmedinf.2019.01.013

[5] S. N. Somani , K. M. Yu , A. G. Chiu , K. J. Sykes , and J. A. Villwock , “Consumer Wearables for Patient Monitoring in Otolaryngology: A State of the Art Review,” Otolaryngology–Head and Neck Surgery 167, no. 4 (2022): 620–631. 34813407 10.1177/01945998211061681 PMC10228711

[6] A. Channa , N. Popescu , J. Skibinska , and R. Burget , “The Rise of Wearable Devices During the COVID‐19 Pandemic: A Systematic Review,” Sensors 21, no. 17 (2021): 5787. 34502679 10.3390/s21175787 PMC8434481

[7] M. Fiore , A. Bianconi , G. Sicari , et al., “The Use of Smart Rings in Health Monitoring: A Meta‐Analysis,” Applied Sciences 14, no. 23 (2024): 10778.

[8] H. S. Kang and M. Exworthy , “Wearing the Future—Wearables to Empower Users to Take Greater Responsibility for Their Health and Care: Scoping Review,” JMIR mHealth and uHealth 10, no. 7 (2022): e35684. 35830222 10.2196/35684 PMC9330198

[9] A. Ehizogie Paul Adeghe , O. Chioma Anthonia Okolo , and O. Olumuyiwa Tolulope Ojeyinka , “A Review of Wearable Technology in Healthcare: Monitoring Patient Health and Enhancing Outcomes,” Open Access Research Journal of Multidisciplinary Studies 7, no. 1 (2024): 142–148.

[10] L. P. Malasinghe , N. Ramzan , and K. Dahal , “Remote Patient Monitoring: A Comprehensive Study,” Journal of Ambient Intelligence and Humanized Computing 10, no. 1 (2019): 57–76.

[11] M. Tanaka , S. Ishii , A. Matsuoka , et al., “Perspectives of Japanese Elders and Their Healthcare Providers on Use of Wearable Technology to Monitor Their Health at Home: A Qualitative Exploration,” International Journal of Nursing Studies 152 (2024): 104691. 38262231 10.1016/j.ijnurstu.2024.104691

[12] J. Pirhonen , L. Lolich , K. Tuominen , O. Jolanki , and V. Timonen , “‘These Devices Have Not Been Made for Older People's Needs’—Older Adults' Perceptions of Digital Technologies in Finland and Ireland,” Technology in Society 62 (2020): 101287.

[13] R. Baines , H. Bradwell , K. Edwards , et al., “Meaningful Patient and Public Involvement in Digital Health Innovation, Implementation and Evaluation: A Systematic Review,” Health Expectations 25, no. 4 (2022): 1232–1245. 35526274 10.1111/hex.13506 PMC9327849

[14] S. M. Kurtz , G. B. Higgs , Z. Chen , et al., “Patient Perceptions of Wearable and Smartphone Technologies for Remote Outcome Monitoring in Patients Who Have Hip Osteoarthritis or Arthroplasties,” Journal of Arthroplasty 37, no. 7 (2022): S488–S492.e2. 35277311 10.1016/j.arth.2022.02.026

[15] S. Madanian , I. Nakarada‐Kordic , S. Reay , and T. Chetty , “Patients' Perspectives on Digital Health Tools,” PEC Innovation 2, no. 2 (2023): 100171. 37384154 10.1016/j.pecinn.2023.100171 PMC10294099

[16] C. Chen , S. Ding , and J. Wang , “Digital Health for Aging Populations,” Nature Medicine 29, no. 7 (2023): 1623–1630. 10.1038/s41591-023-02391-8 37464029

[17] C. Ferguson , L. D. Hickman , S. Turkmani , P. Breen , G. Gargiulo , and S. C. Inglis , “‘Wearables Only Work on Patients That Wear Them’: Barriers and Facilitators to the Adoption of Wearable Cardiac Monitoring Technologies,” Cardiovascular Digital Health Journal 2, no. 2 (2021): 137–147. 35265900 10.1016/j.cvdhj.2021.02.001 PMC8890057

[18] E. P. Garcia Reyes , R. Kelly , G. Buchanan , and J. Waycott , “Understanding Older Adults' Experiences With Technologies for Health Self‐Management: Interview Study,” JMIR Aging 6 (2023): e43197. 36943333 10.2196/43197 PMC10131633

[19] K. Moore , E. O'Shea , L. Kenny , et al., “Older Adults' Experiences With Using Wearable Devices: Qualitative Systematic Review and Meta‐Synthesis,” JMIR mHealth and uHealth 9, no. 6 (2021): e23832. 34081020 10.2196/23832 PMC8212622

[20] M. Abouzahra and M. Ghasemaghaei , “The Antecedents and Results of Seniors' Use of Activity Tracking Wearable Devices,” Health Policy and Technology 9, no. 3 (2020): 213–217.

[21] Z. Ma , Q. Gao , and M. Yang , “Adoption of Wearable Devices by Older People: Changes in Use Behaviors and User Experiences,” International Journal of Human–Computer Interaction 39, no. 5 (2022): 964–987.

[22] J. Wang , D. Carroll , M. Peck , S. Myneni , and Y. Gong , “Mobile and Wearable Technology Needs for Aging in Place: Perspectives From Older Adults and Their Caregivers and Providers,” Studies in Health Technology and Informatics 225 (2016): 486–490. 27332248

[23] T. Schroeder , L. Dodds , A. Georgiou , H. Gewald , and J. Siette , “Older Adults and New Technology: Mapping Review of the Factors Associated With Older Adults' Intention to Adopt Digital Technologies,” JMIR Aging 6 (2023): e44564. 37191976 10.2196/44564 PMC10230357

[24] P. Maresova , E. Javanmardi , S. Barakovic , et al., “Consequences of Chronic Diseases and Other Limitations Associated With Old Age—A Scoping Review,” BMC Public Health 19, no. 1431 (2019): 1431. 31675997 10.1186/s12889-019-7762-5 PMC6823935

[25] M. L. Taylor , E. E. Thomas , K. Vitangcol , et al., “Digital Health Experiences Reported in Chronic Disease Management: An Umbrella Review of Qualitative Studies,” Journal of Telemedicine and Telecare 28, no. 10 (2022): 705–717. 36346938 10.1177/1357633X221119620

[26] H. Kazui , M. Miyajima , E. Mori , and M. Ishikawa . SINPHONI‐2 Investigators . “Lumboperitoneal Shunt Surgery for Idiopathic Normal Pressure Hydrocephalus (SINPHONI‐2): An Open‐Label Randomised Trial,” Lancet Neurology 14, no. 6 (2015): 585–594. 25934242 10.1016/S1474-4422(15)00046-0

[27] M. A. Williams and N. R. Relkin , “Diagnosis and Management of Idiopathic Normal Pressure Hydrocephalus,” Neurology: Clinical Practice 3, no. 5 (2013): 375–385. 24175154 10.1212/CPJ.0b013e3182a78f6b PMC3806933

[28] V. Braun and V. Clarke , “Reflecting on Reflexive Thematic Analysis,” Qualitative Research in Sport, Exercise and Health 11, no. 4 (2019): 589–597.

[29] D. Byrne , “A Worked Example of Braun and Clarke's Approach to Reflexive Thematic Analysis,” Quality & quantity 56 (2022): 1391–1412.

[30] P. Eriksson and A. Kovalainen , Qualitative Methods in Business Research: A Practical Guide to Social Research (Sage, 2015).

[31] H. Tikkanen , K. Heinonen , and A. Ravald , “Smart Wearable Technologies as Resources for Consumer Agency in Well‐Being,” Journal of Interactive Marketing 58, no. 2–3 (2023): 136–150.

[32] T. Pesonen , V. Väisänen , M. Aaltonen , et al., “Determinants of Received Care Time Among Finnish Home Care Clients and Assisted Living Facility Residents: A Time‐Motion Study,” BMC Geriatrics 24, no. 1 (2024): 754. 39266978 10.1186/s12877-024-05355-w PMC11391809

[33] G. Q. Villani , A. Villani , A. Zanni , et al., “Mobile Health and Implantable Cardiac Devices: Patients' Expectations,” European Journal of Preventive Cardiology 26, no. 9 (2019): 920–927. 30823864 10.1177/2047487319830531

[34] E. E. Thomas , M. L. Taylor , A. Banbury , et al., “Factors Influencing the Effectiveness of Remote Patient Monitoring Interventions: A Realist Review,” BMJ Open 11, no. 8 (2021): e051844. 10.1136/bmjopen-2021-051844 PMC8388293 34433611

[35] T. Greenhalgh , J. Wherton , C. Papoutsi , et al., “Beyond Adoption: A New Framework for Theorizing and Evaluating Nonadoption, Abandonment, and Challenges to the Scale‐Up, Spread, and Sustainability of Health and Care Technologies,” Journal of Medical Internet Research 19, no. 11 (2017): e367. 29092808 10.2196/jmir.8775 PMC5688245

[36] J. Sharma , N. Gillani , I. Saied , A. Alzaabi , and T. Arslan , “Patient and Public Involvement in the Co‐Design and Assessment of Unobtrusive Sensing Technologies for Care at Home: A User‐Centric Design Approach,” BMC Geriatrics 25, no. 48 (2025): 48. 39838320 10.1186/s12877-024-05674-y PMC11749497

